# Mammographic features differ with body composition in women with breast cancer

**DOI:** 10.1007/s00330-024-10937-8

**Published:** 2024-07-12

**Authors:** Hanna Sartor, Li Sturesdotter, Anna-Maria Larsson, Ann H. Rosendahl, Sophia Zackrisson

**Affiliations:** 1 Department of Translational Medicine, Diagnostic Radiology, Lund University, Malmö, Sweden; 2 Department of Imaging and Physiology, Skåne University Hospital, Malmö, Sweden; 3 Division of Oncology, Department of Clinical Sciences, Lund University, Lund, Sweden

**Keywords:** Breast, Cancer, Anthropometry, Mammography, Breast density

## Abstract

**Objectives:**

There are several breast cancer (BC) risk factors—many related to body composition, hormonal status, and fertility patterns. However, it is not known if risk factors in healthy women are associated with specific mammographic features at the time of BC diagnosis. Our aim was to assess the potential association between pre-diagnostic body composition and mammographic features in the diagnostic BC image.

**Materials and methods:**

The prospective Malmö Diet and Cancer Study includes women with invasive BC from 1991 to 2014 (*n* = 1116). BC risk factors at baseline were registered (anthropometric measures, menopausal status, and parity) along with mammography data from BC diagnosis (breast density, mammographic tumor appearance, and mode of detection). We investigated associations between anthropometric measures and mammographic features via logistic regression analyses, yielding odds ratios (OR) with 95% confidence intervals (CI).

**Results:**

There was an association between high body mass index (BMI) (≥ 30) at baseline and spiculated tumor appearance (OR 1.370 (95% CI: 0.941–2.010)), primarily in women with clinically detected cancers (OR 2.240 (95% CI: 1.280–3.940)), and in postmenopausal women (OR 1.580 (95% CI: 1.030–2.440)). Furthermore, an inverse association between high BMI (≥ 30) and high breast density (OR 0.270 (95% CI: 0.166–0.438)) was found.

**Conclusion:**

This study demonstrated an association between obesity and a spiculated mass on mammography—especially in women with clinically detected cancers and in postmenopausal women. These findings offer insights on the relationship between risk factors in healthy women and related mammographic features in subsequent BC.

**Clinical relevance statement:**

With increasing numbers of both BC incidence and women with obesity, it is important to highlight mammographic findings in women with an unhealthy weight.

**Key Points:**

- *Women with obesity and BC may present with certain mammographic features*.
- *Spiculated masses were more common in women with obesity, especially postmenopausal women, and those with clinically detected BCs*.
- *Insights on the relationship between obesity and related mammographic features will aid mammographic interpretation*.

## Background

There are several known factors associated with an elevated risk of developing BC (BC). Many of these are related to body composition, hormonal status, and fertility patterns [1, 2]. Body mass index (BMI) is the standard measurement to classify unhealthy weight and obesity [3]. The number of women living with obesity is increasing and is estimated to continue doing so over future decades [4]. A previous review analyzed 82 studies and concluded that obesity was associated with impaired overall survival, as well as BC-specific survival in both pre- and post-menopausal women with BC [5]. In addition, a recent large Danish cohort study with more than 13,000 women enrolled showed an association between obesity and an increased risk of BC recurrence among postmenopausal patients with hormone-receptor-positive early-stage BC treated with aromatase inhibitors [6]. There is also an established link between being overweight in postmenopausal women and the risk of developing BC—particularly hormone receptor-positive BC [7–9].

The potential link between high BMI and specific breast tumor characteristics has been described with mixed findings in the literature. Women with obesity who develop BC have in some studies been described with worse tumor characteristics, such as large tumor size [10] and axillary lymph node involvement [7, 10]. From a radiological perspective, associations between high BMI, worse tumor characteristics, and impaired survival are particularly true for women not participating in mammography screening [11]. This is explained by mammography screening leading to the diagnosis of more tumors at an earlier stage [12, 13]. In contrast, other studies have described associations between BMI and favorable tumor characteristics such as the Luminal A subtype [7, 8] in postmenopausal women with obesity.

The mammographic image is central in the primary diagnosis of BC, in cancer treatment evaluation, and in surveillance programs to detect recurrence. Together with female gender, older age, and family history [2], high breast density is one of the strongest BC risk factors beyond having a high weight [14]. Breast density and BMI are inversely related [15], and these two risk factors have a synergistic effect, meaning a combined risk of high breast density and high BMI that exceeds their individual risk [16, 17]. (Another study did not show such synergistic effects in post-menopausal women [18]). However, these previous studies analyzed density several years before BC diagnosis. Besides density, the mammographic tumor appearance is also associated with certain tumor characteristics and BC subtypes (such as spiculated tumors are more likely to be of luminal A-like subtype) [19], but not with certainty associated with prognosis [20].

An important, but un-answered, clinical question is whether differences in pre-diagnostic body composition will be associated with specific image-related differences in a BC developed years later. From a radiological perspective, if unhealthy body composition is linked to certain mammographic features, then such knowledge could help radiologists focus on certain image features during interpretation. Other body composition measurements such as fat percent and waist circumference may add knowledge beyond BMI. Here, our aim was to assess the potential association between pre-diagnostic body composition and mammographic features in the diagnostic BC image.

## Methods

### Study population

**Fig. 1:**
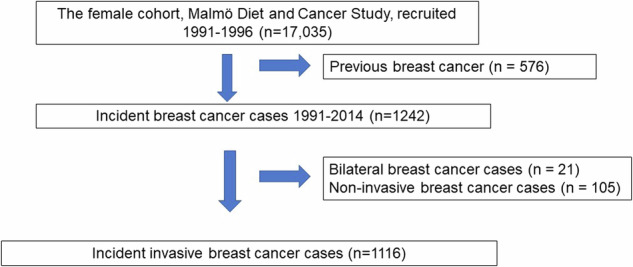
Study population flow chart

This prospective cohort study was performed in accordance with the Declaration of Helsinki and was approved by the Ethics Review Board in Lund, Sweden (official records nos. 652/2005, 166/2007, and 2014/830) and by the Swedish Ethical Review Authority (2022-04473-02). Informed consent was obtained at the baseline. The Malmö Diet and Cancer Study (MDCS) included inhabitants of Malmö from 1991 to 1996 (born 1923–1950) of which 17,035 were women [21, 22]. All women in the cohort with BC diagnosed from 1991 until the end of 2014 were identified. Women with previous BC (*n* = 576), bilateral BC (*n* = 21), or non-invasive cancer (*n* = 105) were excluded (Fig. 1). A total of 1116 women remained eligible for inclusion. The MDCS cohort includes information on vital status, causes and dates of death, and cancer diagnoses, and is updated according to the Swedish Cancer Registry and the Swedish Cause of Death Registry.

### Baseline clinical information

Anthropometric measures were collected at the study baseline examination by study research nurses. Height, weight, waist circumference, and body fat percentage were measured according to the World Health Organization’s definition: BMI in kg/m^2^, waist circumference in centimeters, and body fat in percentage. Patients with a BMI up until 24.9 kg/m^2^ were classified as normal weight, patients with a BMI of 25.0–29.9 kg/m^2^ were classified as overweight, and patients with a BMI of more than 30.0 kg/m^2^ were considered obese. Body composition measures of total body fat mass were recorded using Bioelectrical Impedance Analyzer BIA 103 (RLJ Systems, Clinton Township, MI, USA), with body fat percentage calculated as 100* (fat weight/weight). Information on parity (number of children), ever use of oral contraceptives, menopausal status (peri- and postmenopausal women collapsed in one group when used as an outcome in regression analyses), and use of hormone replacement therapy (HRT) at baseline were recorded at baseline examination.

### Mammographic information

**Fig. 2:**
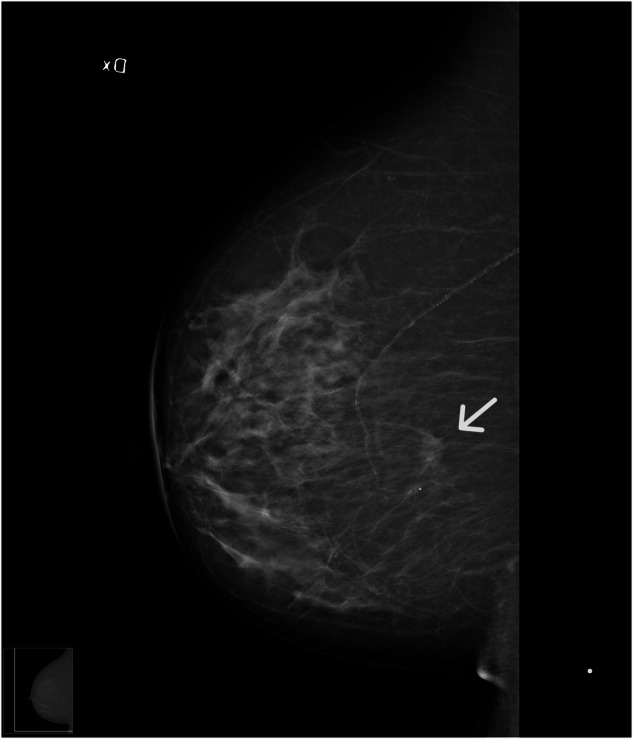
A spiculated mass on mammography (woman with BC, 68-years-old, postmenopausal)

**Fig. 3:**
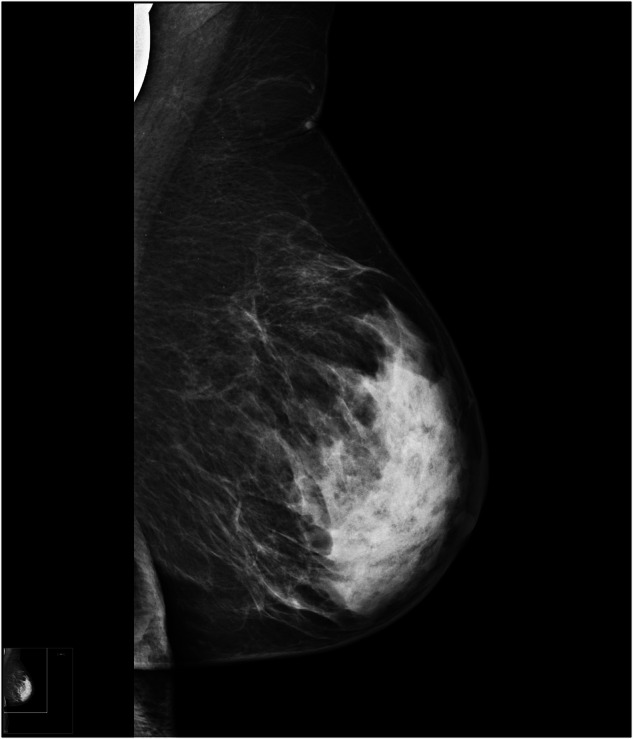
High mammographic density (women with BC in the contralateral breast, 70-years-old, postmenopausal)

Information on the mode of detection (screening or clinical detection), breast density, and mammographic tumor appearance was retrospectively collected from the radiology report at BC diagnosis as described in detail in two recent publications [19, 20]. If the original report (by breast radiologists) was incomplete regarding density and/or tumor appearance, the original mammogram was reviewed retrospectively by authors/radiologists S.Z. and/or H.S. [23]. If the report was inconclusive and the mammogram could not be located, the case was classified as missing (Table 1). Density was recorded as part of the clinical practice, and classified qualitatively using three categories: fat-involuted, moderately dense, and dense breast parenchyma. These groups are represented in the breast imaging reporting and data system (BI-RADS) 4th edition (22): “fat involuted” corresponds approximately to BI-RADS 1 (almost entirely fatty), “moderately dense” corresponds approximately to BI-RADS 2 and 3 (scattered fibroglandular density and heterogeneously dense), and “dense” corresponds approximately to BI-RADS 4 (extremely dense). For the part of the population (*n* = 376) that was diagnosed in 2008–2014, an additional density assessment according to BI-RADS 5th edition was made retrospectively (a = almost entirely fatty, b = scattered areas of fibroglandular density, c = heterogeneously dense, and d = extremely dense). The mammographic tumor appearances were grouped according to an adjusted classification by Luck et al [24]: distinct mass, ill-defined mass, spiculated mass, calcification, and tissue abnormality (a combination of the two groups with architectural distortion and asymmetric tissue component). Examples of mammographic density and tumor appearance, are Figs. 2 and 3.

**Table 1:** Patient characteristics

*n*	1116
Age at diagnosis, years	66 (45, 91)
Missing	0
Parity, *n* (%)	
0	155 (14.2)
1	211 (19.3)
2	504 (46.1)
3	171 (15.6)
4 or more	53 (4.8)
Missing	22
Nullipara	
No	939 (85.8)
Yes	155 (14.2)
Missing	22
Height, m	164.5 (139.0, 180.0)
Missing	0
Weight, kg	68.0 (40.0, 124.0)
Missing	0
Fat weight, percent	31.0 (11.0; 45.0)
Missing	5
Waist, cm	77.0 (55.0; 120.0)
Missing	1
BMI, kg/m^2^	24.95 (16.23, 46.07)
Missing	0
BMI groups, n (%)	
< 25.00	564 (50.5)
25.00–29.99	389 (34.9)
≥ 30	163 (14.6)
Missing	0
Menopause status, *n* (%)	
Pre	337 (30.2)
Peri	93 (8.3)
Post	686 (61.5)
Missing	0
HRT, *n* (%)	
No	823 (73.9)
Yes	290 (26.1)
Missing	3
Oral contraceptives	
No	513 (46.0)
Yes	602 (54.0)
Missing	1
Dominant mammographic tumor features, *n* (%)	
Distinct mass	266 (26.4)
Ill-Defined mass	203 (20.1)
Spiculated	416 (41.2)
Calcifications	83 (8.2)
Tissue abnormality	41 (4.1)
Missing	107
Spiculated mass, *n* (%)	
No	593 (58.8)
Yes	416 (41.2)
Missing	107
Breast density, *n* (%)	
Fat involuted	185 (17.7)
Moderately dense	505 (48.2)
Dense	357 (34.1)
Missing	69
Breast density binary, *n* (%)	
Fat involuted/ moderately dense	690 (65.9)
Dense	357 (34.1)
Missing	69
BI-RADS 5, *n* (%)	
Almost entirely fatty	88 (23.4)
Scattered areas of fibroglandular density	137 (36.4)
Heterogeneously dense	121 (32.2)
Extremely dense	30 (8.0)
Missing	740
BI-RADS 5 binary, *n* (%)	
Almost entirely fatty/ scattered areas of fibroglandular density	225 (59.8)
Heterogeneously dense/extremely dense	151 (40.2)
Missing	740
Mode of detection, *n* (%)	
Clinical	549 (49.7)
Screening	555 (50.3)
Missing	12

Unless otherwise specified data presented as median [range]

### Statistical analyses

Descriptive statistics were used to analyze the study population’s characteristics. All regression analyses were performed for the entire population and then separately by mode of cancer detection and menopausal status, respectively. Crude logistic regression was used to analyze baseline body composition characteristics (BMI (linear and categorical in three groups), waist circumference (continuous), and fat percentage (continuous) in relation to breast density and mammographic tumor appearance. Baseline characteristics known or suspected to be linked with the exposure and the outcome were also included in the linear regression analyses (age at diagnosis (linear), parity (continuous), HRT (binary), oral contraceptives (binary), and menopausal status (categorical)). Breast density was analyzed with the two least dense categories combined (fat involuted + moderately dense parenchyma) versus the densest category (dense breast parenchyma). Analyses were also performed for density assessment according to BI-RADS 5th edition (*n* = 376). These analyses were done using combinations of BI-RADS categories a and b, as well as c and d. Logistic regression analyses regarding density were adjusted for age at diagnosis, BMI, HRT, oral contraceptives, and parity. Logistic regression analyses regarding mammographic tumor appearance were binary: spiculation was one group and other tumor appearances (distinct mass, ill-defined mass, spiculated mass, calcification, and tissue abnormality, a combination of the two groups with architectural distortion and asymmetric tissue component) were the reference group. These analyses were adjusted for breast density and age at diagnosis. Logistic regression yielded odds ratios (ORs) and 95% confidence intervals (CIs). All calculations were performed with R version 4.2.2.

## Results

### Population characteristics

Population characteristics are shown in Table 1. Half of women (564 out of 1116, 50.5%) were of normal weight with a BMI < 25. In addition, most women were postmenopausal at study inclusion (61.5%). In terms of mammographic parameters, the majority (686 out of 1116, 65.9%) of the women had fat-involuted or moderately dense breasts, and half (555 out of 1104, 50.3%) of the cancers were screen-detected. The median time between study inclusion and BC diagnosis was 10.9 years (range 0.02–22.7 years).

### Breast density

Associations between baseline characteristics and anthropometric measures in relation to breast density (univariable analyses) are illustrated in Supplementary Table 1 (full population and clinical mode of breast density measurement) and Supplementary Table 2 (sub-population and breast density according to BI-RADS). Multivariable analyses (Table 2) showed an inverse relation between high breast density and BMI ≥ 30 (OR 0.270 (95% CI: 0.166–0.438), waist circumference (OR 0.948 (95% CI: 0.934–0.963), and fat percentage (OR 0.907 (95% CI: 0.880–0.933)). A similar clear association was seen in analyses stratified for mode of detection and menopausal status with similar inverse relationships obvious in all subgroups. All multivariable analyses on breast density were adjusted for age at diagnosis, HRT, oral contraceptives, and parity.

**Table 2:** Anthropometric measures in relation to the odds ratio of high breast density*

	All		Mode of detection				Menopausal status			
			Clinical		Screening		Pre		Peri/post	
Variable	OR (95% CI)	*p*	OR (95% CI)	*p*	OR (95% CI)	*p*	OR (95% CI)	*p*	OR (95% CI)	*p*
Fat weight, %	0.907 (0.880–0.933)	< 0.001	0.893 (0.855–0.932)	< 0.001	0.925 (0.888–0.964)	< 0.001	0.892 (0.847–0.938)	< 0.001	0.918 (0.885–0.953)	< 0.001
Waist, cm	0.948 (0.934–0.963)	< 0.001	0.941 (0.920–0.963)	< 0.001	0.956 (0.936–0.977)	< 0.001	0.948 (0.922–0.974)	< 0.001	0.950 (0.932–0.968)	< 0.001
BMI groups		< 0.001		< 0.001		< 0.001		0.001		< 0.001
< 25.00	Reference		Reference		Reference		Reference		Reference	
25.00–29.99	0.524 (0.387–0.710)		0.583 (0.380–0.895)		0.493 (0.317–0.768)		0.512 (0.301–0.870)		0.524 (0.359–0.766)	
≥ 30	0.270 (0.166–0.438)		0.201 (0.097–0.418)		0.375 (0.195–0.721)		0.223 (0.085–0.586)		0.303 (0.172–0.534)	

Adjusted for age at diagnosis, HRT, oral contraceptives, and parity

* The table illustrates fat weight, waist circumference, and BMI groups in relation to high breast density (adjusted odds ratio and 95% CI), for all cases and stratified for the mode of detection and menopausal status

### Mammographic tumor appearance

Associations between baseline characteristics and anthropometric measures in relation to the mammographic tumor appearance (spiculated mass) (univariable analyses) are illustrated in Supplementary Table 3. Multivariable analyses (Table 3) showed an association between a high BMI (≥ 30) and a spiculated mass in all women, (OR 1.370 (95% CI: 0.941–2.010)), with an overall *p*-value of 0.007. The association was predominantly observed in women with clinically detected cancer (OR 2.240 (95% CI: 1.280–3.940)) and in postmenopausal women (OR 1.580 (95% CI: 1.030–2.440)). There were no clear associations between waist circumference or fat percent and a spiculated mass among all women, in women with screen-detected cancer, or in analyses stratified on menopausal status. However, women with clinically detected cancer had an association between both higher waist circumference and higher amount of body fat percent and the presence of a spiculated mass. All multivariable analyses on mammographic tumor appearance were adjusted for breast density and age at diagnosis.

**Table 3:** Anthropometric measures in relation to the odds ratio of a spiculated mass on mammography*

	All		Mode of detection				Menopause status			
			Clinical		Screening		Pre		Peri/post	
Variable	OR (95% CI)	*p*	OR (95% CI)	*p*	OR (95% CI)	*p*	OR (95% CI)	*p*	OR (95% CI)	*p*
Fat weight, %	1.020 (0.990–1.050)	0.210	1.070 (1.020–1.110)	0.004	0.977 (0.941–1.010)	0.232	1.000 (0.954–1.050)	0.911	1.030 (0.994–1.060)	0.113
Waist, cm	1.010 (0.995–1.020)	0.237	1.030 (1.010–1.050)	0.004	0.991 (0.974–1.010)	0.325	0.993 (0.969–1.020)	0.604	1.010 (0.999–1.030)	0.067
BMI groups		0.007		0.006		0.050		0.779		0.003
< 25.00	Reference		Reference		Reference		Reference		Reference	
25.00–29.99	0.753 (0.563–1.010)		0.909 (0.583–1.420)		0.617 (0.415–0.918)		0.846 (0.508–1.410)		0.732 (0.513–1.040)	
≥ 30	1.370 (0.941–2.010)		2.240 (1.280–3.940)		0.903 (0.535–1.530)		0.835 (0.368–1.900)		1.580 (1.030–2.440)	

Adjusted for age at diagnosis and breast density

* The table illustrates fat weight, waist circumference, and BMI groups in relation to the presence of a spiculated mass on mammography (adjusted odds ratio and 95% CI), for all cases and stratified for mode of detection and menopausal status

### Combined effect of density and BMI on mammographic tumor appearance

The interaction analysis in Table 4 shows a spiculated mass in relation to breast density and BMI, as well as the interaction between breast density and BMI. Density data suggests that the odds of having a spiculated mass were 24% lower for a woman of normal weight with dense breasts compared to a woman of normal weight with non-dense breasts. BMI data showed that the odds of having a spiculated mass were 22% lower for a woman overweight with non-dense breasts compared to a normal-weight woman with non-dense breasts. In contrast, the odds for a spiculated mass were 36% higher for a woman with obesity and non-dense breasts compared to a woman of normal weight with non-dense breasts. There was no interaction between density and BMI (*p* = 0.879), i.e., the effect of density on the odds of having a spiculated tumor did not differ between the BMI levels.

**Table 4:** Spiculated mass in relation to breast density and BMI, and the interaction between breast density and BMI*

Variable	OR (95% CI)	*p*
Dense	0.759 (0.524–1.097)	0.031
BMI groups		0.007
< 25.00	Reference	
25.00–29.99	0.779 (0.553–1.096)	
≥ 30	1.361 (0.895–2.074)	
Dense × BMI		0.879
< 25.00 and not dense	Reference	
25.00–29.99 and dense	0.869 (0.458–1.630)	
≥ 30 and dense	1.081 (0.408–2.843)	

The interpretation of “Dense” is that the odds of having a spiculated tumor are 24% lower for a normal-weight woman with dense breasts compared to a normal-weight woman with non-dense breasts (note valid for the reference category of BMI)

The interpretation of BMI is that the odds of having a spiculated tumor are 22% lower for an overweight woman with non-dense breasts compared to a normal-weight woman with non-dense breasts. In contrast, the odds for a spiculated mass were 36% higher for a woman with obesity and non-dense breasts compared to a woman of normal weight with non-dense breasts (note valid for the reference category of dense)

Last, we have the interaction effect which is the added effect of having both dense breasts and high BMI

* In the interaction model the interpretation of the main effects is different, i.e., the effect of variable A is valid for the reference category of variable B

## Discussion

This study of 1116 Swedish women with invasive BC from the perspective of MDCS showed associations between unhealthy pre-diagnostic anthropometric measures (BMI, waist circumference, and fat percentage) and a spiculated mass on mammography (OR 1.370 (95% CI: 0.941–2.010), for BMI). We also confirmed the inverse relation between higher anthropometric measures at study inclusion and high breast density at the time of BC diagnosis (OR 0.270 (95% CI: 0.166–0.438), for BMI). There was no evidence of an interaction between breast density and BMI on the risk of having a spiculated mass on mammography.

There is a woman behind every mammogram. Previous research has shown that an unhealthy body composition affects the image in several ways [25]. First, women with obesity are less likely to attend mammography screening [26, 27] or have an ultrasound or magnetic resonance examination performed [28]. Second, breast lumps and lymph nodes may be more challenging to detect clinically in women with obesity and large breasts, [28, 29] and could potentially delay time to diagnosis. This fact highlights the importance of imaging and screening in this population. Third, image quality deteriorates in women with obesity for several reasons [25]. For example, the greater compressed breast thickness may lead to blurred images [30], which may in turn increase the risk of a missed or misinterpreted cancer.

When interpreting and reporting a diagnostic mammogram, the two most important image factors are the breast density and the tumor appearance [31]. Breast density has been a research topic of high interest in recent decades and is an established BC risk factor [32]. Breast density awareness and the implications for the choice of image modality in BC screening have also been debated in the general public [33]. The interplay between breast density, anthropometric measures, and BC, as well as, the potential clinical implications on BC risk models and the screening program—have been extensively studied [17, 34, 35]. In this study, women with higher anthropometric body status (BMI, waist circumference, and fat percentage) years prior to diagnosis have lower breast density at BC diagnosis regardless of age, different menopausal statuses, and different modes of tumor detection, and this time span is a novel contribution to the research field.

Our study found an association between women with obesity and a spiculated tumor appearance. This relationship was most evident in women with clinically detected tumors and postmenopausal women. There is a known clinical link between postmenopausal women with obesity and hormone receptor-positive BC [7] and with hormone receptor-positive BC and a spiculated tumor appearance [19, 36]. Therefore, the results of this complete the chain by adding a scientific link between obesity and the spiculated appearance. On the cellular level, this may be due to the interaction between the cancer cell and adipose tissue, which creates speculations (consisting of tumor cells and stromal tissue) upon mammography, as described by Moriuchi et al [37]. A more fat-involuted breast implies more adipose tissue. The same study supports our findings with an association between higher BMI and spiculated tumors. However, in the present study, the association was not shown for overweight women, which might be a population size issue, but still calls for caution in the interpretation of these findings. It remains unclear whether spiculated tumors are more common in fat-involuted breasts of women with obesity, or if it is merely that the tumors in fat-involuted breasts are more often categorized as spiculated by the radiologists because they are clearly visible to the human eye and not masked by overlapping tissue. A previous paper on the MDCS cohort showed a tendency for women with clinically detected spiculated tumors to have a worse prognosis (adjusted for breast density) [20].

It would have been interesting if the effect of density on the probability of having a spiculated mass on mammography varied as a function of BMI. However, no such interaction could be seen in this study and the study sample size might explain this observation. A previous study showed a positive interaction between density and BMI on BC risk [17], but no previous study ever investigated the effect on mammographic tumor appearance. This is one reason why our study is important.

A major strength of this study was that the population is large and included both clinical and radiological information. However, there are also a few methodological considerations. Women in the MDCS are generally healthier and have a slightly higher educational level than the average female population in Sweden, which may affect the representativeness of the results [21]. In addition, the MDCS is based on data from one single institution, which is also a drawback in terms of generalizability. Nevertheless, the distribution of breast cancer (BC) subtypes, breast density, and mammographic tumor appearances resembles that in everyday Swedish practice and the minor limitations stated above should not affect internal comparisons within the MDCS. Qualitative assessment of mammograms regarding breast density and tumor appearance may be limited by inter-reader variability [38]. Concurrently, this variation reflects everyday clinical practice in a department of breast radiology, which implies that the results may be more generalizable. BMI is a crude, yet clinically easily accessible, measure of adiposity status. As a complement to BMI, total body fat percentage and waist circumference were assessed in relation to breast density and mammographic tumor appearance as more precise measures of overall and central adiposity status, respectively (Supplementary Tables 1–3). The results for these alternative adiposity measures were in line with the associations observed for BMI, in support of the overall findings and conclusions of the study.

It is evident that a woman’s body composition affects the mammographic image. Further research is needed to secure equitable image quality and radiological interpretation for women regardless of body composition. In terms of BC screening strategies and personalized risk assessment, it might be of value to ask for and state the BMI already at the screening appointment. This could make radiographers alert to the potential technical challenges and the radiologist especially alert to minimal signs of speculation when taking care of a woman with a high BMI. In that way, the presence of a clinical risk factor (such as BMI) could assist radiological interpretation, thus making the radiologist suspicious when evaluating a certain mammographic finding and in certain cases performing additional imaging such as breast tomosynthesis or ultrasound.

In conclusion, this study found a positive association between pre-diagnostic obesity and a spiculated mass on mammography at the time of BC diagnosis, predominantly among postmenopausal women and clinically detected tumors. We also showed an inverse relation between obesity at study inclusion and high breast density at the time of BC diagnosis. This study may lead to insights into the relationship between risk factors in healthy women and related mammographic patterns in subsequent BC.

## Supplementary information

supplementary material

## Abbreviations

BC: Breast cancer
BI-RADS: Breast imaging reporting and data system
BMI: Body mass index
HRT: Hormone replacement therapy
MDCS: Malmö Diet and Cancer Study
