# Supplementary material for: Mammographic features differ with body composition in women with breast cancer
Source: Eur Radiol. 2024 Jul 12;35(1):151–9. doi: 10.1007/s00330-024-10937-8 (PMC11632076; doi:10.1007/s00330-024-10937-8)
Supplement: Supplementary file 1 — supplementary material [file 330_2024_10937_MOESM1_ESM.pdf]

Supplementary Table 1 Baseline characteristics in relation to the odds ratio of high breast density

	All		Mode of detection				Menopause status			
			Clinical		Screening		Pre		Peri/Post	
Variable	OR (95% CI)	p	OR (95% CI)	p	OR (95% CI)	p	OR (95% CI)	p	OR (95% CI)	p
Parity	0.738 (0.649-0.839)	<0.001	0.754 (0.631-0.901)	0.002	0.722 (0.599-0.869)	0.001	0.712 (0.561-0.905)	0.010	0.744 (0.638-0.869)	<0.001
Nullipara = No	0.597 (0.419-0.851)	0.004	0.717 (0.438-1.170)	0.187	0.497 (0.298-0.829)	0.007	0.620 (0.319-1.200)	0.158	0.561 (0.367-0.858)	0.008
Height, cm	1.027 (1.004-1.050)	0.019	1.014 (0.9831.047)	0.374	1.041 (1.009-1.075)	0.013	1.036 (0.997-1.077)	0.074	1.014 (0.987-1.043)	0.314
Weight, kg	0.954 (0.941-0.966)	<0.001	0.943 (0.924-0.961)	<0.001	0.964 (0.947-0.982)	<0.001	0.961 (0.939-0.983)	0.001	0.952 (0.936-0.967)	<0.001
Fat weight, %	0.897 (0.873-0.922)	<0.001	0.886 (0.851-0.921)	<0.001	0.906 (0.872-0.942)	<0.001	0.894 (0.851-0.939)	<0.001	0.907 (0.876-0.938)	<0.001
Waist, cm	0.944 (0.930-0.958)	<0.001	0.936 (0.916-0.956)	<0.001	0.950 (0.931-0.970)	<0.001	0.949 (0.924-0.975)	<0.001	0.945 (0.928-0.962)	<0.001
BMI, kg/m^2	0.855 (0.824-0.888)	<0.001	0.832 (0.787-0.880)	<0.001	0.876 (0.832-0.923)	<0.001	0.851 (0.793-0.913)	<0.001	0.864 (0.826-0.904)	<0.001
BMI groups		<0.001		<0.001		<0.001		<0.001		<0.001
<25.00	Reference		Reference		Reference		Reference		Reference	
25.00-29.99	0.457 (0.342-0.610)		0.482 (0.322-0.722)		0.429 (0.283-0.652)		0.526 (0.318-0.870)		0.447 (0.313-0.639)	
≥30	0.249 (0.158-0.392)		0.179 (0.091-0.355)		0.335 (0.182-0.618)		0.228 (0.090-0.580)		0.273 (0.162-0.462)	
Menopause status		<0.001		<0.001		0.707				
Pre	Reference		Reference		Reference					
Peri	0.635 (0.385-1.050)		0.452 (0.206-0.991)		0.810 (0.421-1.560)					
Post	0.590 (0.447-0.779)		0.35 (0.231-0.531)		0.865 (0.586-1.280)					
HRT	1.570 (1.180-2.090)	0.002	1.57 (1.03-2.38)	0.034	1.630 (1.100-2.420)	0.016	0.748 (0.405-1.380)	0.354	2.260 (1.620-3.170)	<0.001
Oral contraceptives	1.520 (1.170-1.970)	0.002	1.79 (1.24-2.58)	0.002	1.340 (0.920-1.940)	0.128	1.490 (0.895-2.500)	0.125	1.320 (0.960-1.810)	0.087

Supplementary Table 2 Baseline characteristics in relation to the odds ratio of high breast density (BI-RADS)

	All		Mode of detection				Menopause status			
			Clinical		Screening		Pre		Peri/Post	
Variable	OR (95% CI)	p	OR (95% CI)	p	OR (95% CI)	p	OR (95% CI)	p	OR (95% CI)	p
Parity	0.716 (0.583-0.880)	0.002	0.807 (0.624-1.040)	0.101	0.570 (0.401-0.812)	0.002	0.586 (0.407-0.844)	0.004	0.784 (0.609-1.010)	0.059
Nullipara = No	0.528 (0.302-0.921)	0.024	0.506 (0.245-1.040)	0.065	0.603 (0.246-1.480)	0.269	0.331 (0.107-1.030)	0.055	0.590 (0.305-1.140)	0.116
Height, cm	0.996 (0.961-1.032)	0.807	1.010 (0.962-1.061)	0.680	0.994 (0.941-1.051)	0.834	1.016 (0.959-1.078)	0.597	0.980 (0.936-1.026)	0.387
Weight, kg	0.966 (0.946-0.985)	<0.001	0.961 (0.936-0.986)	0.002	0.966 (0.934-1.000)	0.049	0.975 (0.944-1.010)	0.120	0.961 (0.936-0.986)	0.003
Fat weight, %	0.957 (0.916-0.999)	0.046	0.917 (0.864-0.974)	0.005	0.987 (0.920-1.060)	0.710	0.959 (0.893-1.030)	0.249	0.958 (0.905-1.010)	0.146
Waist, cm	0.963 (0.942-0.985)	<0.001	0.955 (0.929-0.983)	0.002	0.958 (0.921-0.997)	0.035	0.966 (0.931-1.000)	0.073	0.962 (0.936-0.990)	0.007
BMI, kg/m^2	0.907 (0.857-0.960)	<0.001	0.885 (0.823-0.953)	0.001	0.904 (0.818-0.998)	0.046	0.908 (0.822-1.000)	0.055	0.908 (0.846-0.974)	0.007
BMI groups		0.065		0.038		0.448		0.243		0.290
<25.00	Reference		Reference		Reference		Reference		Reference	
25.00-29.99	0.741 (0.473-1.160)		0.638 (0.349-1.170)		0.762 (0.380-1.530)		0.683 (0.330-1.410)		0.799 (0.449-1.420)	
≥30	0.477 (0.245-0.927)		0.370 (0.167-0.823)		0.473 (0.123-1.820)		0.367 (0.093-1.450)		0.542 (0.248-1.190)	
Menopause status		0.741		0.001		0.164				
Pre	Reference		Reference		Reference					
Peri	0.855 (0.405-1.810)		1.490 (0.353-6.290)		0.737 (0.265-2.050)					
Post	0.844 (0.545-1.310)		0.328 (0.171-0.631)		1.770 (0.866-3.620)					
HRT	1.420 (0.871-2.300)	0.161	1.780 (0.898-3.520)	0.099	1.250 (0.605-2.580)	0.549	1.120 (0.492-2.540)	0.791	1.640 (0.896-3.000)	0.109
Oral contraceptives	1.620 (1.060-2.470)	0.026	2.330 (1.330-4.050)	0.003	1.350 (0.652-2.790)	0.420	2.500 (1.100-5.680)	0.028	1.310 (0.773-2.230)	0.315

Supplementary Table 3 Baseline characteristics in relation to the odds ratio of a spiculated mass on mammography

	All		Mode of detection				Menopause status			
			Clinical		Screening		Pre		Peri/Post	
Variable	OR (95% CI)	p	OR (95% CI)	p	OR (95% CI)	p	OR (95% CI)	p	OR (95% CI)	p
Parity	1.070 (0.944-1.210)	0.295	0.945 (0.785-1.140)	0.544	1.190 (1.000-1.410)	0.048	1.000 (0.795-1.270)	0.973	1.090 (0.947-1.270)	0.222
Nullipara = No	1.300 (0.888-1.890)	0.180	1.060 (0.610-1.830)	0.842	1.520 (0.897-2.560)	0.120	1.110 (0.554-2.240)	0.761	1.370 (0.874-2.150)	0.170
Height, cm	1.011 (0.990-1.033)	0.315	1.010 (0.977-1.045)	0.540	1.009 (0.981-1.039)	0.523	1.002 (0.964- 1.042)	0.909	1.014 (0.988- 1.041)	0.301
Weight, kg	1.010 (0.999-1.020)	0.063	1.030 (1.010-1.040)	0.002	0.998 (0.983-1.010)	0.822	1.000 (0.984-1.030)	0.670	1.010 (1.000-1.030)	0.046
Fat weight, %	1.020 (0.994-1.050)	0.129	1.060 (1.020-1.110)	0.003	0.990 (0.956-1.030)	0.593	1.010 (0.965-1.060)	0.612	1.030 (0.993-1.060)	0.116
Waist, cm	1.010 (0.998-1.020)	0.119	1.030 (1.010-1.050)	0.004	0.998 (0.981-1.010)	0.788	0.998 (0.974-1.020)	0.851	1.030 (0.996-1.060)	0.084
BMI, kg/m^2	1.020 (0.992-1.050)	0.144	1.070 (1.020-1.120)	0.003	0.987 (0.947-1.030)	0.546	1.010 (0.953-1.080)	0.675	1.010 (1.000-1.030)	0.041
BMI groups		0.007		0.006		0.127		0.945		0.002
<25.00	Reference		Reference		Reference		Reference		Reference	
25.00-29.99	0.799 (0.605-1.060)		0.948 (0.618-1.450)		0.704 (0.484-1.020)		0.918 (0.558-1.510)		0.767 (0.546-1.080)	
≥30	1.470 (1.020-2.120)		2.210 (1.300-3.760)		1.060 (0.641-1.770)		0.974 (0.435-2.180)		1.660 (1.090-2.520)	
Menopause status		0.680		0.815		0.969				
Pre	Reference		Reference		Reference					
Peri	1.030 (0.635-1.680)		1.150 (0.508-2.610)		0.967 (0.528-1.770)					
Post	0.898 (0.680-1.190)		0.921 (0.588-1.440)		1.030 (0.716-1.490)					
HRT	1.140 (0.857-1.520)	0.367	0.994 (0.638-1.550)	0.978	0.855 (0.605-1.210)	0.374	1.360 (0.751-2.470)	0.309	1.100 (0.793-1.530)	0.562
Oral contraceptives	0.937 (0.729-1.210)	0.615	0.925 (0.633-1.350)	0.686	1.230 (0.837-1.790)	0.296	1.010 (0.606-1.700)	0.956	0.879 (0.650-1.190)	0.405
